# Stretching Mucins: Exploring the Complex Rheology of a Natural Glycoprotein Network

**DOI:** 10.1021/acsmacrolett.6c00270

**Published:** 2026-08-21

**Authors:** Bianca Hazt, George D. Degen, Lucas Warwaruk, Daniel J. Read, Adam O’Connell, Oliver G. Harlen, Gareth H. McKinley, Anwesha Sarkar

**Affiliations:** † Food Colloids and Bioprocessing Group, School of Food Science and Nutrition, 4468 University of Leeds, Woodhouse Lane, Leeds LS2 9JT, United Kingdom; ‡ Department of Mechanical Engineering, 2167 Massachusetts Institute of Technology, 77 Massachusetts Avenue, Cambridge, Massachusetts 02139, United States; § Polymer Science Platform, 544023 Reckitt Benckiser Healthcare (U.K.) Ltd., Dansom Lane S, Hull HU8 7DS, United Kingdom; ∥ School of Mathematics, 4468 University of Leeds, Woodhouse Lane, Leeds LS2 9JT, United Kingdom

## Abstract

Flow and extensional deformation of mucin networks are fundamental in mucus biophysics, governing how mucus functions as a protective, lubricating, and transport-facilitating layer. While the shear and oscillatory rheology of mucin solutions have been characterized in considerable detail, their behavior under extensional deformation remains comparatively understudied. Here, we report a concentration-dependent transition in the extensional flow response of mucin solutions using a bespoke dripping-onto-substrate extensional rheometer. We show that mucin solutions at the lower concentrations undergo linear filament thinning, whereas semidilute mucin solutions form highly extensible filaments, with radius decaying exponentially in time, consistent with the elastocapillary thinning observed in solutions of high molecular weight synthetic polymers. Remarkably, at higher mucin concentrations interchain mucin associations produce a sudden reduction in the apparent elastocapillary relaxation time. We demonstrate how increasing macromolecular concentration redistributes the balance between viscous and elastic stresses during capillary thinning in a biopolymer network and reveal a concentration-driven reduction in mucin filament extensibility.

## Graphical abstract

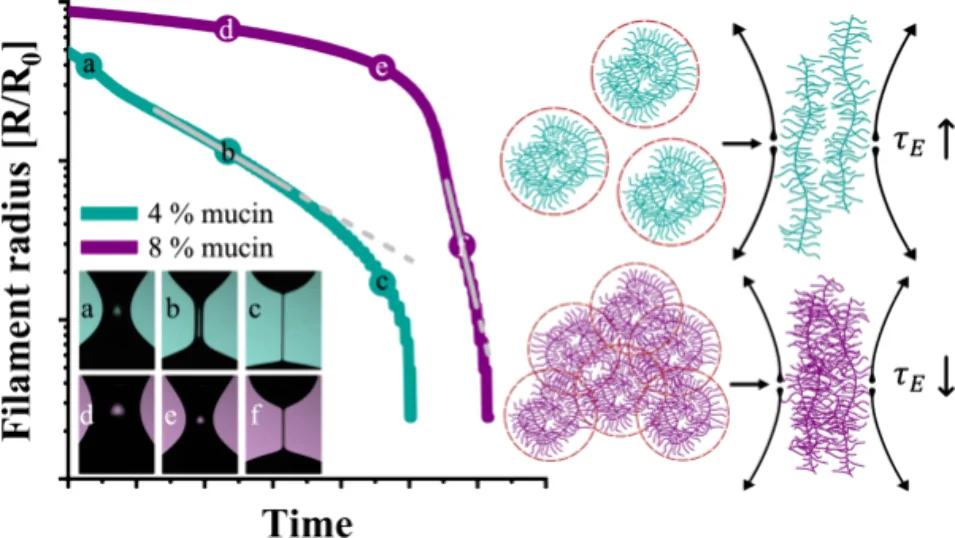

Mucus is a biological hydrogel essential for ocular, respiratory, gastrointestinal, and reproductive health. Its complex rheological behavior impacts its ability to lubricate surfaces, facilitates clearance of undesirable particulates or microorganisms, and regulates transport and microbial populations at mucosal surfaces.^1,2^ The material properties of mucus arise primarily from gel-forming mucin glycoproteins assembled into supramolecular disulfide-linked networks, and from their interactions with other minor mucosal components.^3^ Variations in mucin concentration, which *in vivo* span nearly 2 orders of magnitude, may lead to distinct mechanical responses under physiological stresses. In different physiological settings, this tunability serves distinct (and sometimes undesirable) roles. Highly elastic, weakly extensible mucus is required to form a robust cervical mucus plug during a healthy pregnancy,^4,5^ whereas excessively viscoelastic mucus can impair transport and clearance in diseases such as cystic fibrosis or chronic obstructive pulmonary disease.^6,7^ Concentration-dependent changes in the shear rheology of mucus and mucin solutions are well documented,^8–11^ as well as in the dynamics of filament thinning for saliva.^12^ However, how mucin concentration regulates the extensional response of mucin solutions, which is central to filament breakup, airway clearance, and transport through constricted geometries, has not been established. While there have been studies on hagfish slime and its associated glycoprotein fraction,^13,14^ and of native biological fluids such as saliva and bronchial sputum,^15^ extensional measurements of reconstituted mammalian mucin solutions remain scarce, with existing reports limited to unpurified gastric mucin-containing formulations examined at concentrations up to 4 wt %.^16^

In this letter, we investigate purified mucin solutions over concentrations ranging from 0.25 to 8 wt %. Small-amplitude oscillatory shear (SAOS) is first used to define the concentration-dependent linear viscoelastic response, followed by measurements of extensional capillarity-driven filament thinning using dripping-onto-substrate (DoS) rheometry.^17,18^ With increasing concentration, a qualitative transition in the extensional behavior is observed at concentrations corresponding to the formation of a critical gel. This transition is characterized by increased elasticity and dynamic viscosity, which delay the formation of an extensional filament in DoS, and a reduction in the apparent relaxation time extracted from the elastocapillary regime.

Bovine submaxillary mucin (BSM, Sigma-Aldrich), a widely used and well-characterized model mucin system,^8^ was purified by extensive dialysis against ultrapure water^19^ to remove nonmucin components. The resulting mucin exhibits a radius of gyration *R*_g_ = 165 nm and weight-average molecular weight *M̅*_w_ = 39.3  MDa, as previously determined by AF4-MALS,^20^ yielding an estimated overlap concentration c* = 0.34 wt %,^20^ and intrinsic viscosity [η] = 227 mL g^–1^ using the Graessley relation.^21^ All mucin solutions were prepared in 10 mM HEPES buffer at pH 7.0.

We measured the linear viscoelastic moduli using a strain-controlled ARES-G2 (TA Instruments), equipped with a 2° 40 mm cone-and-plate geometry; a 2 wt % Tween 80 layer and a mineral oil overlay were deposited at the air–liquid interface to prevent interfacial effects and evaporation, respectively. The storage and loss moduli *G*′(ω), *G*′′(ω) for 2, 4, and 5 wt % BSM are characteristic of the low frequency/terminal regime of a viscoelastic liquid (*G*″ ∝ ω, *G*′ ∝ ω^2^). Although the measurements are restricted to this low-frequency limit, we can extrapolate the data to estimate a characteristic relaxation where *G*′ = *G*″. For 2, 4 and 5 vol % BSM, this gives relaxation time estimates of ∼1.6, ∼2.7, and 4.3 ms, respectively (Figure 1; Figure S1, Supporting Information), consistent with unentangled viscoelastic behavior on the time scales probed. For *c*/c* ≪ 1, the longest relaxation time approaches a constant Zimm relaxation time at infinite dilution,^22^ τ_*Z*_ ∼ η_*s*_ *R*_g_^3^/(*k*_B_ *T*), which is around 1 ms using *R*_g_ determined from AF4-MALS. Therefore, the increase in relaxation time with concentration is attributable to intermolecular interactions above the overlap concentration c*. In this regime, chains are no longer isolated and relax within a progressively crowded medium, in which the effective resistance to motion increases due to the presence of surrounding polymers.^23,24^

**Figure 1:**
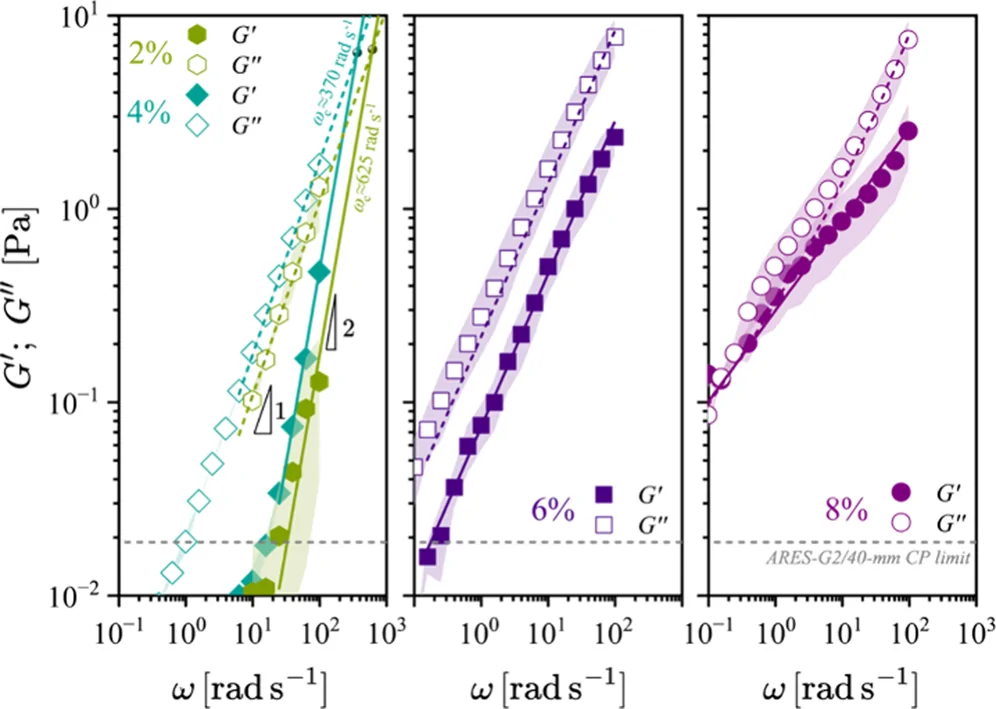
Dynamic viscoelastic response of BSM solutions obtained from small amplitude oscillatory shear frequency sweeps at 2, 4, 6, and 8 wt %. Mean values ± standard deviation (shaded regions) are shown for triplicate measurements. Solid and dashed lines represent fits to *G*′ and *G*″, respectively. Fits correspond to a Maxwell model (2 and 4 wt %), the Scott Blair model for a critical gel with β ≈ 0.81 (6 wt %), and a fractional Kelvin–Voigt liquid (6 wt %; with fitted parameters $V=0.054{\mathrm{Pa\cdot s}}^{\alpha },G=0.334{\mathrm{Pa\cdot s}}^{\beta },\alpha =1,\beta =0.45$ ).

Figure 1 shows that further increasing the BSM concentration to 6 and 8 wt % leads to a pronounced change in the linear viscoelastic response. At 8 wt %, both moduli increase with frequency by a common power, with a nearly constant phase angle tan δ = *G*″/*G*′ over the measured frequency range, consistent with the Winter–Chambon criterion for a critical gel.^25^ This response can be represented by the fractional Scott Blair spring-pot model, $\sigma \left(t\right)=G\left[d^{\beta }\gamma \left(t\right)\right]/\left[dt^{\beta }\right]$ , for which tan δ = tan­(πβ/2) is frequency independent. Fitting this model to the 6 wt % data yields β ≈ 0.81. At 8 wt %, elasticity increases further and tan δ now increases with frequency, taking a value close to unity at the lowest measured frequencies (Figure S1, Supporting Information), indicating that the material becomes increasingly gel-like and the response departs from a single spring-pot description. The response of the 8 wt % solution can be represented by a fractional Kelvin–Voigt liquid model, previously employed for weakly viscoelastic biopolymer networks such as unpurified BSM;^8^ fitting details and evidence that the elasticity arises from intermolecular interactions are provided in the Supporting Information (Tables S1 and S2, Figures S1 and S2). Additional SAOS measurements at 10 and 14 wt % further support this concentration-dependent increase in elasticity, with *G*′ > *G*′′ and tan δ < 1 over the measured frequency range (Supporting Information, Table S3 and Figure S1).

The extensional properties of mucin solutions were measured using DoS. In this technique, an unstable liquid bridge forms when a pendant drop contacts a partially wetting substrate, enabling characterization of weakly elastic materials with submillisecond relaxation time scales.^26^ A syringe pump (PHD 2000, Harvard Apparatus) was used to form a drop through a blunt-end nozzle of radius *R*_0_ = 0.635 mm. The substrate was brought into contact with the droplet at a fixed distance *H* = 4.4*R*_0_ below the nozzle. The neck was imaged at *f*_s_ = 16500 Hz using a high-speed camera (NOVA S20, Photron), a high-magnification zoom lens (LAOWA, Venus Optics), and a light emitting diode (SOLIS-525C, Thorlabs Inc.) for backlighting. Following Warwaruk et al.,^26^ we determine the neck radius as the axial minimum, *R*(*t*) = min­{*R*(*z*,*t*)}, where *R*(*z*,*t*) is the filament radius at axial position *z* and time *t*. Figure 2 shows the evolution of *R*(*t*)/*R*_0_ on linear and semilogarithmic axes (Figure 2a,b), the corresponding apparent extensional strain rate, ε̇(*t*) (Figure 2c), and representative high-speed image sequences (Figure 2d–h) for HEPES buffer and 0.25–8 wt % BSM. Replicated measurements are presented in Figure S3 (Supporting Information).

**Figure 2:**
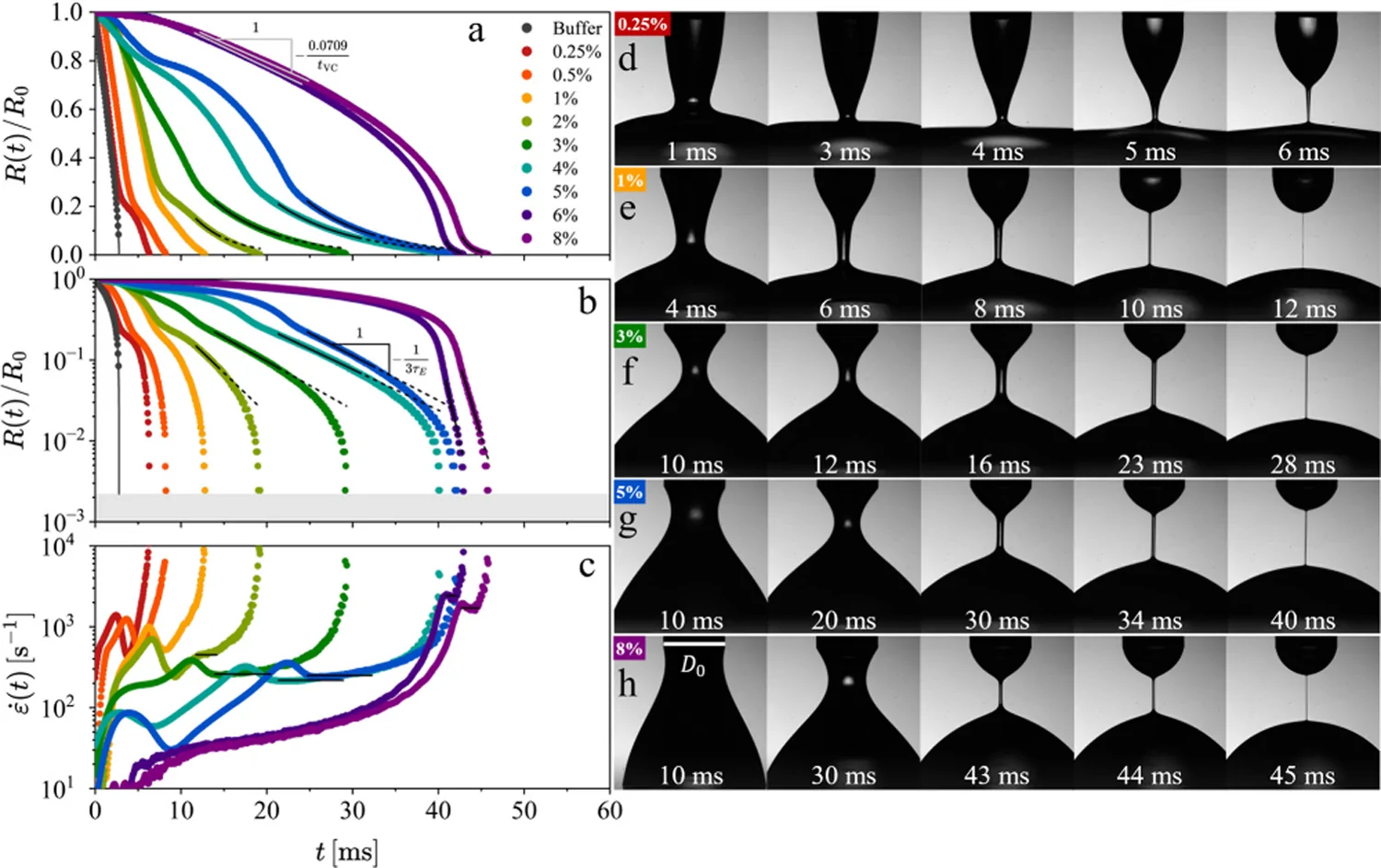
Temporal evolution of the normalized minimum filament radius *R*(*t*)/*R*_0_ in capillary thinning for BSM solutions from 0.25 wt % to 8 wt %, and for 10 mM HEPES buffer at pH 7.0. (a) Data presented on a linear–linear axis highlight the overall thinning behavior across concentrations, with a VC balance established at intermediate times in the 6 and 8 wt % BSM solutions indicated by a linear fit. (b) The same profiles shown on semilogarithmic axes (logarithmic ordinate, linear abscissa) reveal the emergence of an exponential decay regime at concentrations *c* ≥ 2 wt % characteristic of EC thinning. The gray shaded region indicates the minimum measurable radius *R*_res_ = 1.51 μm, corresponding to one pixel. (c) The extensional rate ε̇(*t*) extracted from the radius evolution plotted as a function of time. For each plot, the time interval over which a constant extension rate is established [and from which the characteristic relaxation time is obtained] is marked with solid black lines and extended by black dashed lines. Representative high-speed image sequences illustrate the capillarity-driven filament thinning and final breakup of the BSM solutions for (d) 0.25 wt %, (e) 1 wt %, (f) 3 wt %, (g) 5 wt %, (h) 8 wt %. The scale bar in (h) represents the nozzle diameter *D*_0_ = 2*R*_0_ = 1.27 mm.

There are several distinct regimes for capillarity-driven thinning depending on the balance between the capillary pressure, inertial, viscous, and elastic contributions to the stress. When viscous and elastic stresses are negligible, inertia and surface tension govern inertio-capillary (IC) thinning: *R*(*t*)/*R*_0_ = *A*[(*t*_b_ – *t*)/*t*_R_]^2/3^, where *t*_b_ is the breakup time, *t*_R_ is the Rayleigh time scale, *t*_R_ = (ρ*R*_0_^3^/Γ)^1/2^, with surface tension Γ (see Supporting Information, Table S4, Figure S4) and fluid density ρ, and *A* is a system-dependent prefactor satisfying 0.4 ≤ *A* ≤ 1.^27^ This expression describes the thinning dynamics of the HEPES buffer, as shown in Figure 2a,b for *A* ≈ 0.73. Figure S5 (Supporting Information) shows that the filament has an axially asymmetric and cone-shaped morphology prior to breakup.^28^

More viscous Newtonian fluids exhibit visco-capillary (VC) thinning, with *R*(*t*)/*R*_0_ = 0.0709­(*t*_b_ – *t*)/*t*_VC_, where *t*_VC_ = η*R*_0_/Γ is the viscous time scale.^29^ The separation between IC and VC scalings is governed by the Ohnesorge number Oh = *t*_VC_/*t*_R_.^30,31^ For Oh ≪ 1, inertia dominates, and thinning follows IC scaling, whereas for Oh ≥ 1, viscous stresses dominate, and samples exhibit VC thinning.^32,33^ For our BSM solutions, Oh increases from O ­(10^–3^) at the lowest concentration to Oh ≈ 0.78 at the highest concentration (Supporting Information, Table S5), indicating a transition from inertia-dominated to increasingly viscously dominated initial filament thinning conditions (see fits for 6 and 8 wt % BSM, Figure 1a).

For viscoelastic fluids, the filament thinning may initially follow either IC or VC scalings (depending on the background viscosity) until elastic stresses become significant and the thinning dynamics crossover to elasto-capillary (EC) balance.^34^ In this regime, *R*(*t*) is predicted to decay exponentially with time:

1 $$\frac{R\left(t\right)}{R_{0}}\approx {\left(\frac{GR_{0}}{2\Gamma }\right)}^{1/3}\exp\left(-\frac{t-t^{*}}{3\tau_{E}}\right)$$

Here, *G* is the elastic modulus of the fluid and t* is the experimentally observed time at which the flow transitions to EC thinning and a cylindrical filament forms (Figure 2e–h). By fitting the observed exponential decay to eq 1, an effective extensional relaxation time *τ*_*E*_ can be extracted. This EC regime holds until the mucin chains in solution reach their limit of finite extensibility and the filament then transitions to a terminal finitely extensible nonlinear elastic regime.^35–37^

For 0.25 and 0.5 wt % BSM (Figure 2d), the filament forms a short-lived, slender filament that undergoes capillary breakup before an axially uniform filament can be established. Although this response indicates elastic effects, the filament is too short-lived to establish a well-defined EC regime. By contrast, the 1–5 wt % BSM solutions produce axially uniform filaments (e.g. Figure 2e, image at 10 ms) that undergo exponential thinning consistent with eq 1 (denoted by black lines in Figure 2a,b). This elasto-capillary regime can also be seen in Figure 2c, which shows the time evolution of the local extensional strain rate ε̇(*t*) at the minimum radius

2 $$\mathrm{\varepsilon ̇}\left(t\right)\equiv -\frac{2}{R\left(t\right)}\frac{dR\left(t\right)}{dt}$$

In the EC regime the extensional strain rate approaches a constant value of *ε̇*_EC_ = 2/(3τ_*E*_). This is preceded by an inertio-elastic overshoot,^38,39^ characterized by a rapid increase and sharp decrease in ε̇(*t*), previously associated with a coil–stretch transition.^36,40^ For BSM concentrations between 2 and 5 wt % both the onset time and the duration of the exponential thinning regime increase with concentration consistent with the behavior of increasingly viscoelastic synthetic polymer solutions.^41^ For 6 and 8 wt % BSM, filament thinning is qualitatively different (Figure 2a–c): ε̇(*t*) increases monotonically and the radius decreases approximately linearly in time for the first 30 ms, consistent with VC thinning, followed by a markedly short-lived EC regime.

The tensile stress growth coefficient (or apparent extensional viscosity) η_*E*_^+^(*t*) = Δσ_*E*_(*t*)/ε̇(*t*) can be evaluated from sufficiently time-resolved measurements of the minimum filament radius *R*(*t*) and is agnostic to specific models for filament thinning dynamics (e.g., VC or EC thinning). Figure 3 shows η_*E*_^+^(*t*) as a function of the time-evolving capillary pressure Δσ_*E*_(*t*) = Γ/*R*(*t*) for 0.25–5  wt % (Figure 3a) and 6–8  wt % BSM (Figure 3b). Shaded regions indicate limits within which η_*E*_^+^ and Δσ_*E*_ should not be interpreted due to experimental limitations; these are detailed by Warwaruk et al.^26^ and also shown in Figure S6 (Supporting Information). As the filaments thin, all samples exhibit stress thickening within the EC regime (Figure 3, filled symbols), with η_*E*_^+^(*t*) increasing approximately linearly with Δσ_*E*_(*t*) on log–log axes, consistent with an elasto-capillary stress balance. For 6 and 8 wt % BSM, an initial apparent extensional-thinning region precedes the EC transition at Δσ_*E*_(*t*) ≈ 10^3^ Pa. The magnitude of η_*E*_^+^(*t*) increases from 1 to 5 wt % BSM but then decreases at 6–8 wt % despite the larger tensile stresses achieved, indicating a change in the characteristic extensional relaxation dynamics.

**Figure 3:**
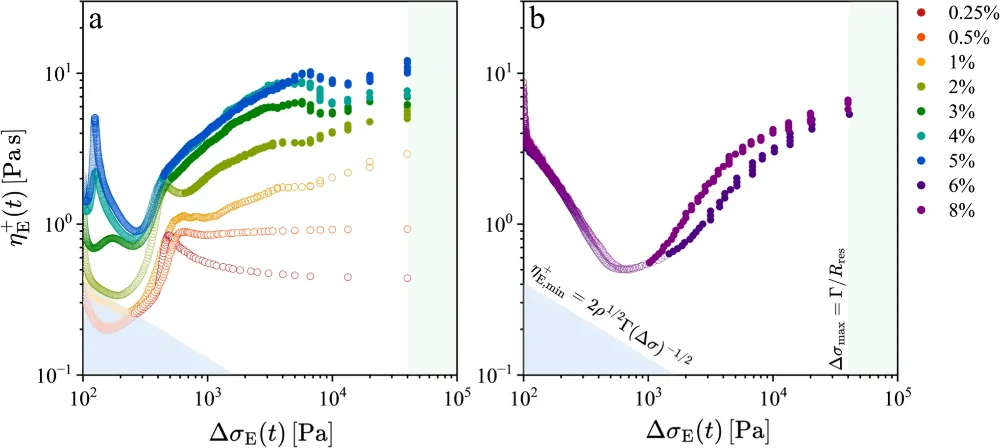
Apparent extensional viscosity (or tensile stress growth coefficient) as a function of the time-evolving extensional stress difference for BSM solutions: (a) 0.25–5 wt % and (b) 6 and 8 wt %. Open symbols denote pre-elastocapillary (EC) regimes; filled symbols denote data at and beyond the transition to EC thinning. The colored shaded regions denote areas in which measurements of η_*E*_^+^ and Δ*σ*_*E*_ become unreliable; these corresponding limits are described in further detail in Figure S6 of the Supporting Information.

The effective relaxation times, τ_*E*_, extracted from EC thinning are shown in Figure 4 as a function of BSM concentration. Up to 4  wt %, the relaxation times obtained from the extrapolated SAOS data and from DoS experiments agree within experimental uncertainty (Supporting Information, Table S6), suggesting that the shear and extensional responses are governed by the same longest relaxation time. However, at 5  wt %, the relaxation time obtained from DoS is significantly lower than the linear relaxation time. For 6 and 8  wt % BSM, relaxation times extracted from the EC regime are substantially lower than those measured for *c* ≤ 5  wt %. This abrupt change has not been previously reported for mucin solutions.

**Figure 4:**
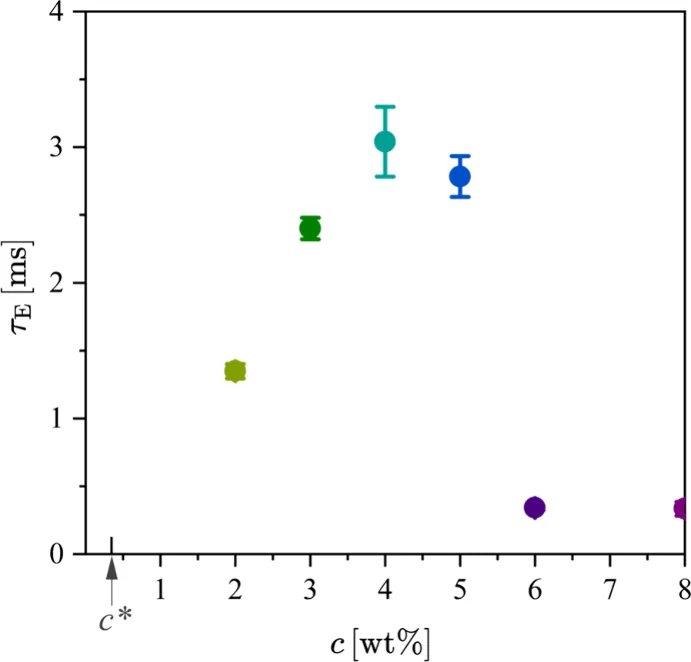
Extensional relaxation times extracted from the elastocapillary (EC) regime for each BSM concentration at which an EC regime was observed. The estimated overlap concentration c* for BSM is indicated on the graph. Symbols and error bars represent the mean ± standard deviation of five independent measurements.

The relationship between τ_*E*_ and the linear viscoelastic relaxation time τ has been shown to depend on macromolecular extensibility for model DNA.^42^ For highly extensible molecules, τ_*E*_ ≈ τ, while for polymers with smaller extensibility a reduced ratio τ_*E*_/τ is observed.^42^ Here, we observe a precipitous decrease in τ_*E*_ as BSM concentration increases. While changes in concentration do not alter the intrinsic extensibility of the individual mucin chains, they do modify the bulk extensional flow response of the fluid filament and the conditions under which elastic stresses become dominant. Numerical simulations of capillarity-driven thinning for entangled polymer systems described by the Doi–Edwards-Marrucci-Grizzuti (DEMG) and Rolie-Poly (RP) models predict a delayed transition to the EC regime compared to semidilute systems.^43^ For these entangled systems, with increasing constraint density, the transition to the EC regime is expected to happen only close to filament breakup.^43^ However, this crossover happens gradually with increasing polymer concentration beyond *c*_*e*_, as entanglement effects become important, and does not explain the precipitous transition observed here for a physically associated biopolymer network.

Similar to observations in entangled polymer solutions,^43–45^ the 6 and 8 wt % BSM samples exhibit a delayed onset of the EC regime, a reduced τ_*E*_, and no clear signature of a coil–stretch transition. However, unlike classical entangled polymers, the linear viscoelastic response of these BSM samples does not exhibit a well-defined plateau modulus, but instead displays the signatures of a weakly physically associated network. The concentrated BSM samples are therefore better described as transient, weakly elastic networks in which intermolecular associations impose constraints that limit filament extensibility. In this sense, while the DEMG/RP models attribute reduced extensibility to an increased number of entanglements per chain, a similar macroscopic response emerges here from transient physical associations, leading to a shortened elasto-capillary regime, lower effective molecular extensibility, and a reduction in τ_*E*_. This comparison with synthetic and model polymer solutions is intended to help identify common signatures of capillarity-driven filament thinning, such as delayed EC onset and reduced τ_*E*_, but does not establish that these characteristics share a molecular origin.

To visualize qualitatively these concentration-dependent changes in the microstructure of BSM solutions, samples were chemically fixed with glutaraldehyde, subjected to critical point drying, and then imaged by Scanning Electron Microscopy following established protocols.^46,47^ At 2 wt %, no material remained adhered to the glass substrate after drying, indicating the absence of any fixed or percolated network able to withstand preparation. At intermediate concentrations (5 and 6 wt %, Figure S7, Supporting Information), some BSM remained attached to the substrate, suggesting emerging intermolecular associations. At 8 wt %, the dehydrated BSM exhibited a continuous, porous gel-like morphology (Figure 5), with multiscale connectivity evident throughout the structure. Under identical preparation conditions, only the higher-concentration samples formed a continuous structure. Although the apparent pore sizes are likely influenced by drying and should not be overinterpreted, the interconnected network observed only at higher mucin concentration is consistent with the onset of concentration-driven physical association and with the reduced magnitudes of the extensional responses observed in the DoS measurements.

**Figure 5:**
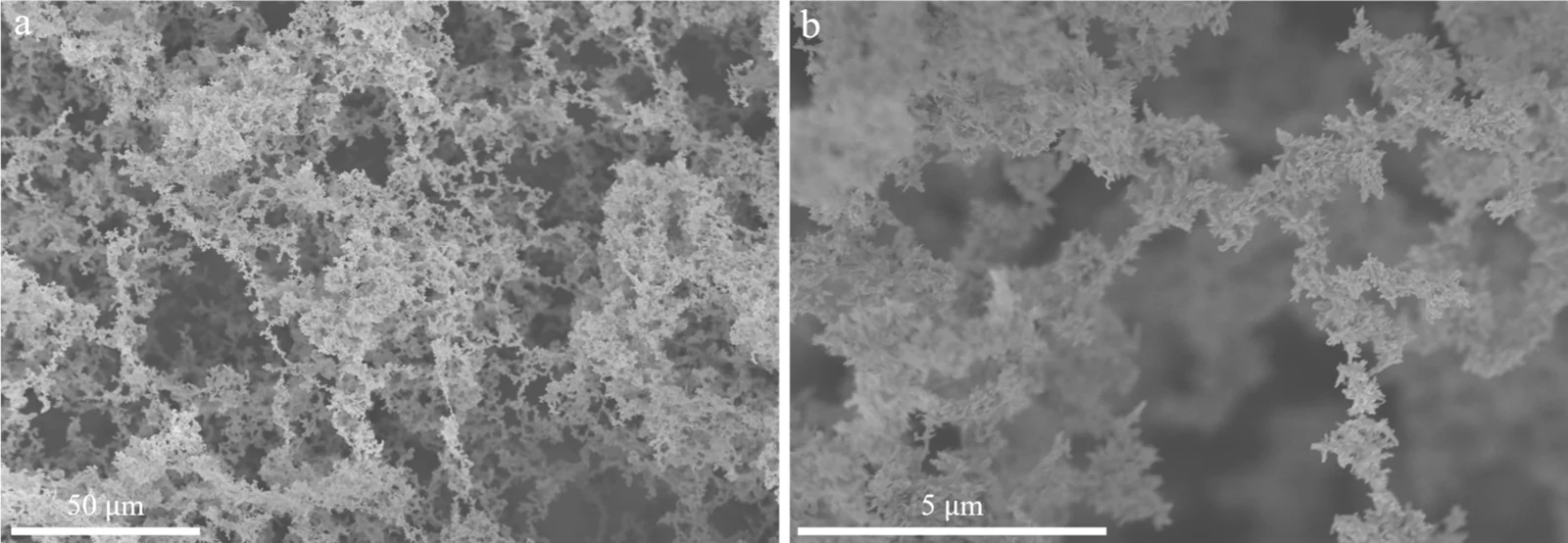
Representative scanning electron microscopy (SEM) images obtained for an 8 wt % BSM sample. Scale bars represent (a) 50 μm and (b) 5 μm.

In conclusion, we have shown that both the shear and the extensional responses of mucin solutions are highly sensitive to concentration. Amid ongoing efforts to understand polymer interactions under extensional flows,^44^ these results identify concentrated mucin solutions as a distinct class of weakly extensible, transiently interconnected networks. Semidilute mucin solutions form highly extensible filaments, whereas more concentrated samples are dominated by viscous contributions to the tensile stress difference and enter the elastocapillary regime only at smaller filament radii. The concentration range of 0.25 – 8 wt % spans physiologically and pathologically relevant mucus environments, including conditions in which mucin overproduction contributes to altered airway mucus rheology and disease severity. It should be noted that the purified mucin solutions in our study do not include the many minor mucosal components that are known to modify mucus rheology.^3^ Further rheological studies systematically incorporating these components into model mucin systems will help bridge the gap toward understanding the concentration-dependent nonlinear behavior of fully constituted mucus. We envision that this work may contribute to new diagnostic strategies for assessing mucus rheology, and guide the design of bioinspired materials with tunable “stringiness”, i.e., resistance to capillarity-driven filament thinning mediated by concentration, solvent interactions and intermolecular interactions.

## Supplementary Material



## Notes

Mathematical details of fractional rheological models; SAOS characterization and viscoelastic model fits across concentrations, including tan δ analysis (Figure S1); Steady shear and chemical perturbation effects (Figure S2); Surface tension measurement; Filament thinning dynamics and Bond number effects (Figures S3–S5); Filament capture rate (FCR) analysis; Tensile stress growth coefficient–stress relationships with limit lines (Figure S6); SEM images for BSM at 5 and 6 wt % (Figure S7); Ohnesorge number, surface tension and relaxation time comparisons (Tables S4–S6) (PDF)

## #

G.D.D.: School of Chemical and Biomolecular Engineering, Georgia Institute of Technology, North Avenue, Atlanta, Georgia 30332, United States
