# Supplementary material for: Stretching Mucins: Exploring the Complex Rheology of a Natural Glycoprotein Network
Source: ACS Macro Lett. 2026 Aug 21;15(9):1215–21. doi: 10.1021/acsmacrolett.6c00270 (PMC13585127; doi:10.1021/acsmacrolett.6c00270)
Supplement: Supplementary file 1 [file mz6c00270_si_001.pdf]

Supporting Information

Stretching mucins: exploring the complex rheology of a natural glycoprotein network

Bianca Hazt, [†,‖,****] George D. Degen, [‡] Lucas Warwaruk, [‡] Daniel J. Read, [‖] Adam O'Connell, [§] Oliver G. Harlen, [‖,***] Gareth H. McKinley, [‡,**] Anwesha Sarkar, [†,*]

[†] Food Colloids and Bioprocessing Group, School of Food Science and Nutrition, University of Leeds, Woodhouse Lane, Leeds LS2 9JT, United Kingdom

[‡] Department of Mechanical Engineering, Massachusetts Institute of Technology, 77 Massachusetts Avenue, Cambridge, Massachusetts 02139, United States

[§] Polymer Science Platform, Reckitt Benckiser Healthcare (UK) Ltd, Dansom Lane S, Hull HU8 7DS, United Kingdom

[‖] School of Mathematics, University of Leeds, Woodhouse Lane, Leeds LS2 9JT, United Kingdom

**** Email: mmbh@leeds.ac.uk; *** Email: o.g.harlen@leeds.ac.uk; ** Email: gareth@mit.edu: *Email: a.sarkar@leeds.ac.uk

Table of contents

Fractional rheological models S-4

Table S1 — Summary of the Maxwell and fractional mechanical models used in this work, their constitutive relations, characteristic relaxation timescale, and values of $\tan\delta$ from the oscillatory frequency response S-4

Table S2 — Fitted quasi-properties ($\mathbb{V}$, $\mathbb{G}$) and power law exponents (α, β) of the fractional Kelvin-Voigt liquid model for BSM samples at 6 and 8 wt%. Reported values represent mean values ± standard deviation for independent fits to triplicate measurements. S-5

Table S3 — Fitted quasi-properties ($\mathbb{V}$, $\mathbb{G}$) and power law exponents (α, β) for the fractional Scott Blair critical gel model for BSM samples at 10 wt% and 14 wt%. Reported values represent mean values ± standard deviation for independent fits to triplicate measurements S-6

Figure S1 — (a) Linear viscoelastic response of BSM at 2, 4, and 5 wt%. Extrapolation of $G'' \sim \omega$ and $G' \sim \omega^2$ for BSM at 5 wt% yields an estimated $G' = G''$ crossover at 232 rad s^{-1} ($\tau \simeq 0.0043$s). (b) Fractional Kelvin-Voigt liquid (FKV-Liquid) model fits for 6 and 8 wt% BSM (fitted parameters are reported in Table S2). (c) Fractional Scott Blair (Spring-pot) fit for BSM at 10 wt% and 14 wt%. (d) Frequency dependence of $\tan\delta$. Solid lines represent a single-mode Maxwell fit where $\tan\delta = 1/(\tau\omega)$ for 4 and 5 wt% BSM, a critical-gel fit for BSM at 6, 10 and 14 wt%, where $\tan\delta = \tan(\pi\beta/2) = G''/G'$; an FKV liquid model fit for BSM at 8 wt% where $\tan\delta = [\mathbb{V}\omega + \mathbb{G}\omega^\beta \sin(\pi\beta/2)]/[\mathbb{G}\omega^\beta \cos(\pi\beta/2)]$... S-8

Figure S2 — (a) Steady-shear viscosity as a function of shear rate, measured using a 40 mm cone-and-plate geometry (2° CP-40 mm). The minimum rheometer sensitivity is shown as a grey dashed line. BSM samples at 8, 4, 2, 1, and 0.5 wt% were prepared in 10 mM HEPES buffer at pH 7.0. Consistent with the emergence of a strong concentration-dependent linear viscoelastic response, these steady shear viscosity measurements reveal pronounced shear-thinning behavior for 8 wt% BSM. (b) Viscoelastic behavior of 8 wt% BSM in the absence or in the presence of 8 M urea, or 20 mM dithiothreitol (DTT). Shaded regions represent the standard deviations of three independent measurements. In the presence of DTT, which reduces disulfide bonds between mucin subunits, the covalent network at 8 wt% is disrupted, restoring a terminal liquid-like response with $G' \sim \omega^2$ and $G'' \sim \omega$, consistent with the Maxwell description. The corresponding relaxation time is $\tau = 8.60$ ms. In contrast, 8 wt% BSM in the presence of 8 M urea, which perturbs hydrogen bonding and weak non-covalent associations without reducing disulfide linkages, does not recover terminal behaviour of a viscoelastic liquid: while $G'' \sim \omega$ is preserved, the elastic modulus G' exhibits an apparent $G' \sim \omega^{1.5}$ scaling over the accessible frequency window. (c) Steady-shear viscosity of the untreated 8 wt% BSM sample shown as a function of shear rate, compared with the complex viscosity extracted from G' and G'', shown as a function of angular frequency, for 8 wt% BSM in the presence of either 8 M urea or 20 mM DTT S-9

Figure S3 — Plots of the normalized filament radius $R(t)/R_0$ as a function of $(t_b - t)$, where t_b is the time to breakup. For each concentration, at least three independent videos are included. Independent measurements performed using a second high-speed imaging setup (operating at an image acquisition rate of $f_s = 2{,}095$ Hz) and separately prepared BSM solutions reproduced the same concentration-dependent thinning behavior. In the terminal stage of the filament lifetime, replicates at a given concentration superimpose, and the trajectories for all concentrations converge as the filament approaches breakup. For completeness, data are presented using (a) log-linear, (b) log-log, and (c) linear-linear axes. The inset in (c) shows the terminal region on linear-linear axes S-10

Figure S4 — Surface tension (Γ, mN m^{-1}) of BSM at 2 wt% as a function of time (s). Mean $\pm$ standard deviation from triplicate measurements is shown. Across all concentrations (0.25-8 wt%), the initial surface tension is indistinguishable within experimental resolution. Deviations only emerge at longer timescales, beyond the temporal window relevant to DoS measurements ($<$ 1 s). Inset: variation of surface tension over 1-4 s, highlighting negligible early-time variations. S-11

Terminal capillarity-driven thinning behavior of dilute BSM samples S-12

Figure S5 — Dripping-on-substrate measurements with $D_0 = 2R_0 = 1.27$ mm (a) for the solvent used in this work [10 mM HEPES buffer at pH 7.0 (blue)]. The data for 10 mM HEPES buffer at pH 7.0 are shown for the profile extracted from images performed at a Bond number $Bo = 0.35$. Snapshots of 10 mM HEPES buffer at pH 7.0 at a separation distance that yield Bond numbers of (b) $Bo = 0.51$ and (c) $Bo = 0.35$. The conical shape is most clearly observed at smaller needle-substrate separation distances H, corresponding to lower Bond numbers ($Bo = R_0 H\rho g/\Gamma$), as gravitational effects are reduced relative to capillarity at the moment the pendant drop touches the substrate. For the buffer, the Ohnesorge number $Oh = \eta_0/\sqrt{\rho\Gamma R_0}$ is $Oh \approx 4.4 \times 10^{-3}$, placing the system well within the inertio-capillary regime. The time to breakup (t_b) for HEPES buffer is 2.78 ms. (d) Normalized minimum filament radius $R(t)/R_0$ as a function of $t_b - t$ for 0.25 and 0.5 wt% BSM. In the final stage of thinning, both samples approach $R(t)/R_0 \sim (t_b - t)^1$ as indicated by the slope $n = 1$, consistent with a viscosity-dominated terminal thinning regime. (e-f) Corresponding high-speed image sequences for (e) 0.25 wt% ($t_b = 6.24$ ms) and (f) 0.5% BSM ($t_b = 8.11$ ms)................... S-13

Filament capture rate (FCR) analysis S-14

Figure S6 — Apparent transient tensile stress growth coefficient plotted as a function of the time-evolving extensional stress difference for BSM dispersions spanning 1 to 8 wt%. Limit lines, as discussed by Warwaruk *et al.*[3], are drawn using $\rho = 1080$ kg m^{-3}, $\Gamma = 61.7$ mN m^{-1}, $R_0 = 0.635$ mm, $R_{res} = 1$ pixel $= 1.51 \times 10^{-6}$ m, $FCR_0 = f_s \ln m_0$, with $f_s = 16500$ s^{-1} and $m_0 = R_0/R_{res} = 430.46$. The horizontal lines shown in (a) indicate the asymptotic values of the apparent transient tensile stress growth coefficient asymptotic transient $\eta_{E,\infty}$, as previously discussed (see the discussion on page S-12).............................. S-15

Figure S7 — Representative Scanning Electron Microscopy images of BSM samples prepared at (a) 6 wt% and (b) 5 wt%. Scale bars represent 10 µm S-16

Table S4 — Measured surface tension using a pendant drop tensiometer (OCA 25, DataPhysics Instruments, Germany). Drops (22 µL) were formed at the tip of a 1.65 mm DS500 GT syringe, and the liquid-air surface tension was obtained by fitting the drop profile to the Young-Laplace equation using dpiMAX software. A density of 1080 kg m^{-3} was assumed in the fitting procedure. Measurements were performed in triplicate for each concentration; the reported value include the standard deviation. The initial surface tension was used in all capillarity-driven thinning analyses, as filament thinning occurs on timescales much shorter than those required for interfacial equilibration.. S-17

Table S5 — Reference Ohnesorge number ($Oh = \eta_0/\sqrt{\rho\Gamma R_0}$) for the BSM concentrations investigated. Here, $\rho = 1080$ kg m^{-3}, $\Gamma = 61.7$ mN m^{-1}, $R_0 = 0.635$ mm. The reported value of η_0 is an estimate from the low-shear viscosity as the zero-shear plateau was not directly resolved in steady shear.................................. S-17

Table S6 — Comparison between relaxation times (τ) obtained from SAOS estimated from the extrapolated crossover frequency for 2-5 wt% (and from $\tau = (\mathbb{V}/\mathbb{G})^{1/(1-\beta)}$ at 8 wt%), and the effective relaxation times extracted from elastocapillary (EC) thinning regimes in DoS extensional experiments (τ_E) for BSM across concentrations. The time to breakup (t_b) for each fluid is also shown.. S-17

References.. S-17

Fractional rheological models

As discussed in the main text, the linear viscoelastic response of BSM samples at concentrations up to 5 wt% can be fitted to a Maxwell model, which consists of a linear mechanical combination of a Hookean spring and a dashpot in series. In this model, the resulting viscoelastic stress relaxation is governed by a single timescale τ, which can be obtained from the crossover between power-law slopes of 2 and 1 for the storage and loss moduli, respectively[1]. For the higher-concentration samples that did not display $G' \propto \omega^2$ and $G'' \propto \omega$ ($c > 5$ wt%), the experimental (G') and (G'') data were fitted simultaneously using a set of fractional constitutive models, including the spring-pot, fractional Maxwell and fractional Kelvin-Voigt models and their limiting liquid and gel forms, and the fractional Zener model. Model selection was based on the Bayesian information criterion,

$$BIC = \left(N_f + 1\right)\ln(n) + n\,[\ln(2\pi) + 1 + \ln(\sigma^2)],$$

with $\sigma^2 = RSS/n$, where N_f is the number of fitted model parameters, n is the total number of G' and G'' residuals, and RSS is the corresponding sum of squared residuals. For each dataset, the best-fitting and most credible model was identified as the model with the lowest BIC value among the candidate fits. The selected constitutive models are summarised in Table S1.

Table S1 – Summary of the Maxwell and fractional mechanical models used in this work, their constitutive relations, characteristic relaxation timescale, and expressions for $\tan\delta$ from the oscillatory frequency response.

	Constitutive relation	Relaxation timescale τ	$\tan\delta$
Maxwell model 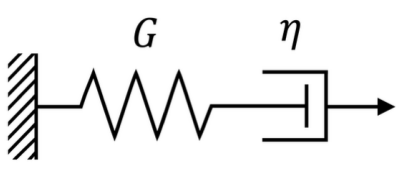	$\sigma(t) + \frac{\eta}{G}\frac{\mathrm{d}\sigma(t)}{\mathrm{d}t} = \eta\frac{\mathrm{d}\gamma(t)}{\mathrm{d}t}$	$\tau = \frac{\eta}{G}$	$\tan\delta = \frac{G''}{G'} = \frac{1}{\tau\omega}$
Spring-pot 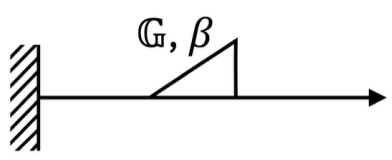	$\sigma(t) = \mathbb{G}\frac{\mathrm{d}^\beta\gamma(t)}{\mathrm{d}t^\beta}$	-	$\tan\delta = \tan\left(\frac{\pi\beta}{2}\right)$
Fractional Kelvin-Voigt liquid model 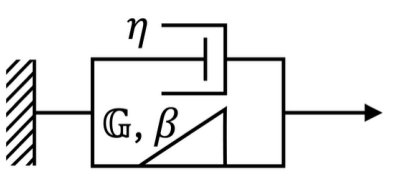	$\sigma(t) = \eta\frac{\mathrm{d}\gamma(t)}{\mathrm{d}t} + \mathbb{G}\frac{\mathrm{d}^\beta\gamma(t)}{\mathrm{d}t^\beta}$	$\tau = \left(\frac{\eta}{\mathbb{G}}\right)^{\frac{1}{1-\beta}}$	$\tan\delta = \frac{\eta\omega + \mathbb{G}\omega^\beta\sin(\pi\beta/2)}{\mathbb{G}\omega^\beta\cos(\pi\beta/2)}$

Spring-pot and FKV-liquid responses at 6 and 8 wt% BSM

Table S1 summarises the constitutive relation, the expected relaxation time and expression for $\tan\delta$ for a spring-pot (also known as the Scott Blair fractional mechanical model) that describes a power-law response at the critical gel point, with a quasi-property $\mathbb{G}$ and power-law exponent β. Building from the spring-pot definition, the Fractional Kelvin-Voigt Model (FKVM) consists of two spring-pot elements arranged in a parallel configuration, with quasi-properties $\mathbb{V}$ and $\mathbb{G}$, and exponents α and β. The stress evolution for the FKV model is therefore represented by the expression

$$\sigma(t) = \mathbb{V}\frac{\mathrm{d}^\alpha\gamma(t)}{\mathrm{d}t^\alpha} + \mathbb{G}\frac{\mathrm{d}^\beta\gamma(t)}{\mathrm{d}t^\beta}.$$

For the 8 wt% sample, the FKV-liquid model produced the lowest BIC and was therefore selected. For the 6 wt% sample, the simpler spring-pot produced the lowest BIC. The 6 wt% data were also fitted using the FKV-liquid model to examine how the two descriptions are related. The FKVL model is a limiting case of the FKVM in which $\alpha = 1$. The fitted parameters for the 6 and 8 wt% BSM samples using the FKV-liquid model are summarised in Table S2.

Table S2 – Fitted quasi-properties ($\mathbb{V}$, $\mathbb{G}$) and power law exponents (α, β) of the fractional Kelvin-Voigt liquid model for BSM samples at 6 and 8 wt%. Reported values represent mean values ± standard deviation for independent fits to triplicate measurements.

	α	$\mathbb{V}$ (Pa s$^{\alpha}$)	β	$\mathbb{G}$ (Pa s$^{\beta}$)
6 wt%	1	0.0316 ± 0.027	0.73 ± 0.08	0.258 ± 0.01
8 wt%	1	0.0543 ± 0.014	0.45 ± 0.02	0.334 ± 0.02

For the FKV-liquid model, the loss modulus is

$$G'' = \mathbb{V}\omega + \mathbb{G}\omega^{\beta}\sin(\pi\beta/2).$$

Reduction to a Scott Blair element requires the viscous dashpot term to remain negligible throughout the experimental window, i.e.

$$\mathbb{V}\omega \ll \mathbb{G}\omega^{\beta}\sin(\pi\beta/2).$$

Enforcing this condition at the highest measured frequency, ω_{max} , gives

$$\omega_{max} \ll \left(\frac{\mathbb{G}\sin(\pi\beta/2)}{\mathbb{V}}\right)^{\frac{1}{1-\beta}}.$$

Using the fitted parameters shown in Table S2, this characteristic frequency ω_{max} is approximately 1.69×10^{3} rad s^{-1} for the 6 wt% sample, and 12.4 rad s^{-1} for the 8 wt% sample. Consequently, for the 6 wt% sample, the condition $\omega_{max} \ll \omega_c$ is satisfied across the entire experimental window, such that $\mathbb{V}\omega \ll \mathbb{G}\omega^{\beta}$ and the viscous dashpot contribution becomes negligible. In this regime, the FKV-liquid constitutive equation reduces to

$$\sigma(t) \approx \mathbb{G}\frac{\mathrm{d}^{\beta}\gamma(t)}{\mathrm{d}t^{\beta}},$$

which corresponds to a single spring-pot element with exponent β and quasi-property $\mathbb{G}$, consistent with the definition in Table S1:

$$\sigma(t) = \mathbb{G}\frac{\mathrm{d}^{\beta}\gamma(t)}{\mathrm{d}t^{\beta}}.$$

The measured response is therefore governed by a single power-law, yielding an approximately frequency-independent tan δ, consistent with critical-gel like behavior within the experimental window. By contrast, for the 8 wt% sample, ω_c lies within the measured frequency range, such that the constraint $\mathbb{V}\omega \ll \mathbb{G}\omega^{\beta}$ is not satisfied at higher frequencies. Both the dashpot and fractional contributions are therefore important within the experimental window, and the full FKV-liquid description is required.

Having shown that the 6 wt% sample lies in the asymptotic regime of the FKV-liquid model where the viscous contribution is negligible, the data were fitted with a Scott Blair constitutive form, from which the fractional

exponent β was obtained and the corresponding frequency-independent value of the phase angle, $\tan\delta = \tan(\pi\beta/2)$, was calculated.

Spring-pot model: 6, 10 and 14 wt% samples

Additional 10 and 14 wt% samples were also tested to see if further increasing the concentration would enhance the elastic contribution to the linear viscoelastic response. The corresponding fitted quasi-properties and power-law exponent values for these two samples are summarised in Table S3.

Table S3 – Fitted quasi-properties ($\mathbb{V}$, $\mathbb{G}$) and power law exponents (α, β) for the fractional Scott Blair critical gel model for BSM samples at 10 wt% and 14 wt%. Reported values represent mean values ± standard deviation for independent fits to triplicate measurements.

	β	$\mathbb{G}$ (Pa s$^{\beta}$)
6 wt%	0.81 ± 0.021	0.25 ± 0.06
10 wt%	0.30 ± 0.032	9.36 ± 0.05
14 wt%	0.31 ± 0.012	18.30 ± 2.48

The marked decrease in β from 6 to 10 wt%, followed by its similar value at 14 wt%, indicates a transition towards a more elastic response, with little further change in frequency dependence at higher concentrations. The main change between 10 and 14 wt% is the magnitude of the quasi-property $\mathbb{G}$, which characterises the strength of the viscoelastic response and increases by approximately 96%. At 14 wt%, a slight low-frequency deviation from the model is observed (Figure S1). This may indicate that the system is approaching its solubility limit or may reflect a transient physical network whose bonds rearrange over sufficiently long timescales, rather than a permanently crosslinked gel with a true equilibrium plateau modulus. Distinguishing between these possibilities is beyond the scope of this study.

Overall concentration-dependent small-amplitude oscillatory rheological behaviour of BSM

The emergence of increasingly elastic behavior at higher mucin concentrations suggests that elastic stresses arise from a growing density of transient physical crosslinks between mucin macromolecules. These associations increasingly constrain chain motion, while remaining able to rearrange over sufficiently long timescales and therefore not producing a finite equilibrium plateau modulus. As mentioned in the main text, a two-element, four-parameter fractional Kelvin-Voigt model has previously been used to describe the linear viscoelastic behavior of weakly viscoelastic biopolymer networks, including unpurified BSM. In the present work, this constitutive representation also produced the lowest BIC among the tested models for the 8 wt% sample. The transition from 6 to 8 wt% is characterized by a decrease in the fractional exponent β, which describes the frequency dependence of the viscoelastic response, together with an increase in the quasi-property $\mathbb{G}$, which reflects the strength of the fractional contribution. These changes indicate an increasing elastic transient network contribution as the mucin concentration is raised. For the 6 wt% sample, the viscous contribution of the FKV-liquid model becomes negligible over the measured frequency range, such that the model reduces to the single spring-pot form. Consistently, the Scott Blair/spring-pot model produced the lowest BIC for this concentration and described the nearly common power-law dependence of G' and G'', with an approximately frequency-independent $\tan\delta$, despite $G'' > G'$.

The 8 wt% sample represents an intermediate regime in which both the dashpot and fractional contributions are apparent within the experimental frequency window, requiring the full FKV-liquid description. Further increasing the mucin concentration to 10 and 14 wt% enhances the elastic response, producing substantial

increases in both G' and G'', with $G' > G''$ and $\tan\delta < 1$ throughout the measured frequency range. At these concentrations, the response again approaches a single power law described by the spring-pot model, but with a substantially lower exponent than at 6 wt%, consistent with the formation of a stronger and more elastic physical network. The similar values of the exponent β at 10 and 14 wt% indicate little further change in the frequency dependence of the response, whereas the increase in $\mathbb{G}$ indicates continued strengthening of the network. Overall, increasing the mucin concentration from 6 to 14 wt% leads to systematic increases in G' and G'' and a reduction in $\tan\delta$, reflecting a transition from a predominantly dissipative response towards a more elastic, physically connected network. At 14 wt%, the slight low-frequency deviation from the spring-pot model may indicate that the system is approaching its solubility limit or may reflect slow rearrangement within a transient physical network.

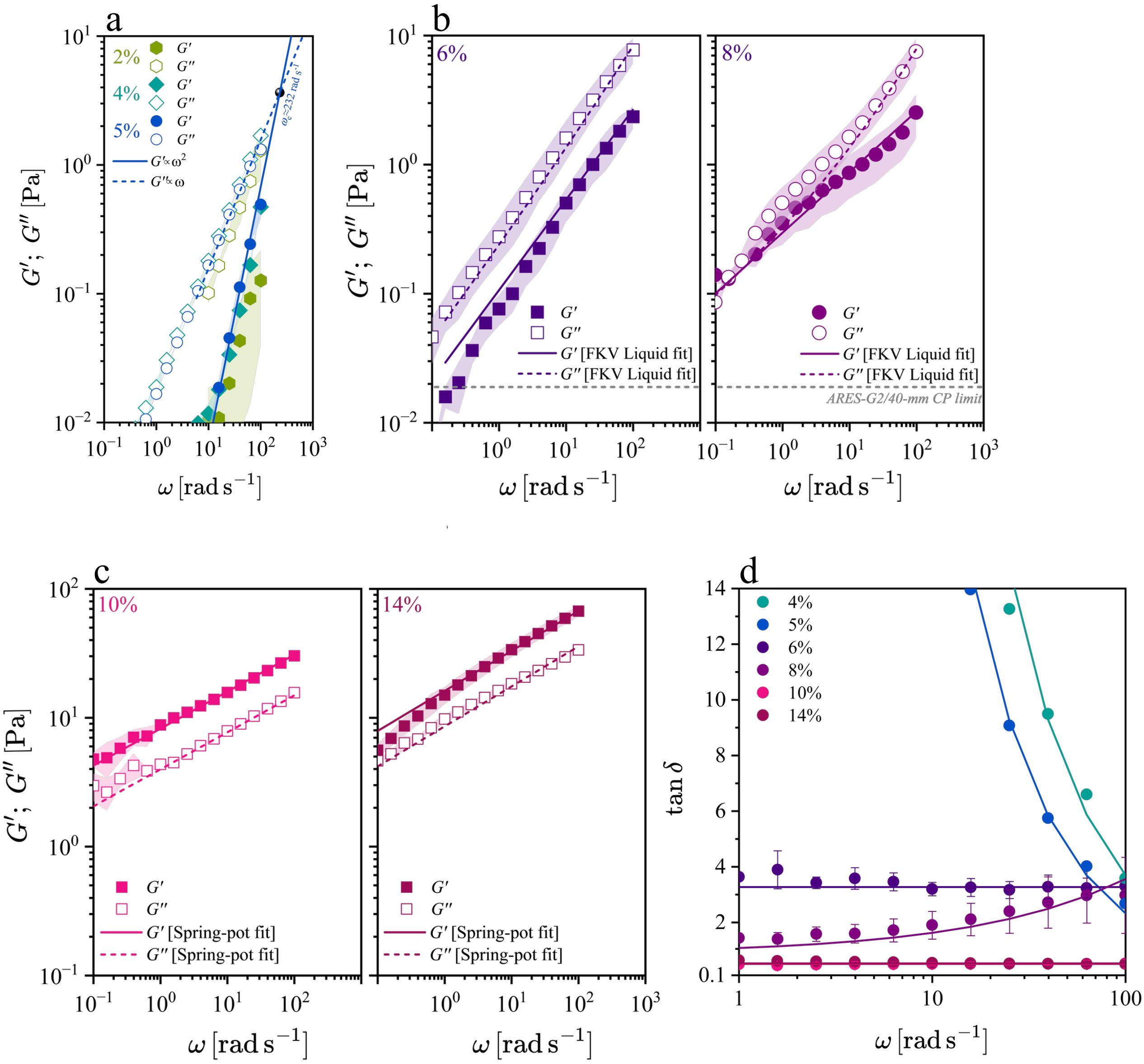

Figure S1 – (a) Linear viscoelastic response of BSM at 2, 4, and 5 wt%. Extrapolation of $G'' \sim \omega$ and $G' \sim \omega^2$ for BSM at 5 wt% yields an estimated $G' = G''$ crossover at 232 rad s^{-1} ($\tau \simeq 0.0043$s). (b) Fractional Kelvin-Voigt liquid (FKV-liquid) model fits for 6 and 8 wt% BSM (fitted parameters are reported in Table S2). (c) Fractional Scott Blair (spring-pot) fit for BSM at 10 wt% and 14 wt%. (d) Frequency dependence of $\tan\delta$. Solid lines represent a single-mode Maxwell fit where $\tan\delta = 1/(\tau\omega)$ for 4 and 5 wt% BSM, a critical-gel fit for BSM at 6, 10 and 14 wt%, where $\tan\delta = \tan(\pi\beta/2) = G''/G'$; an FKV-liquid model fit for BSM at 8 wt% where $\tan\delta = [\mathbb{V}\omega + \mathbb{G}\omega^\beta \sin(\pi\beta/2)]/[\mathbb{G}\omega^\beta \cos(\pi\beta/2)]$.

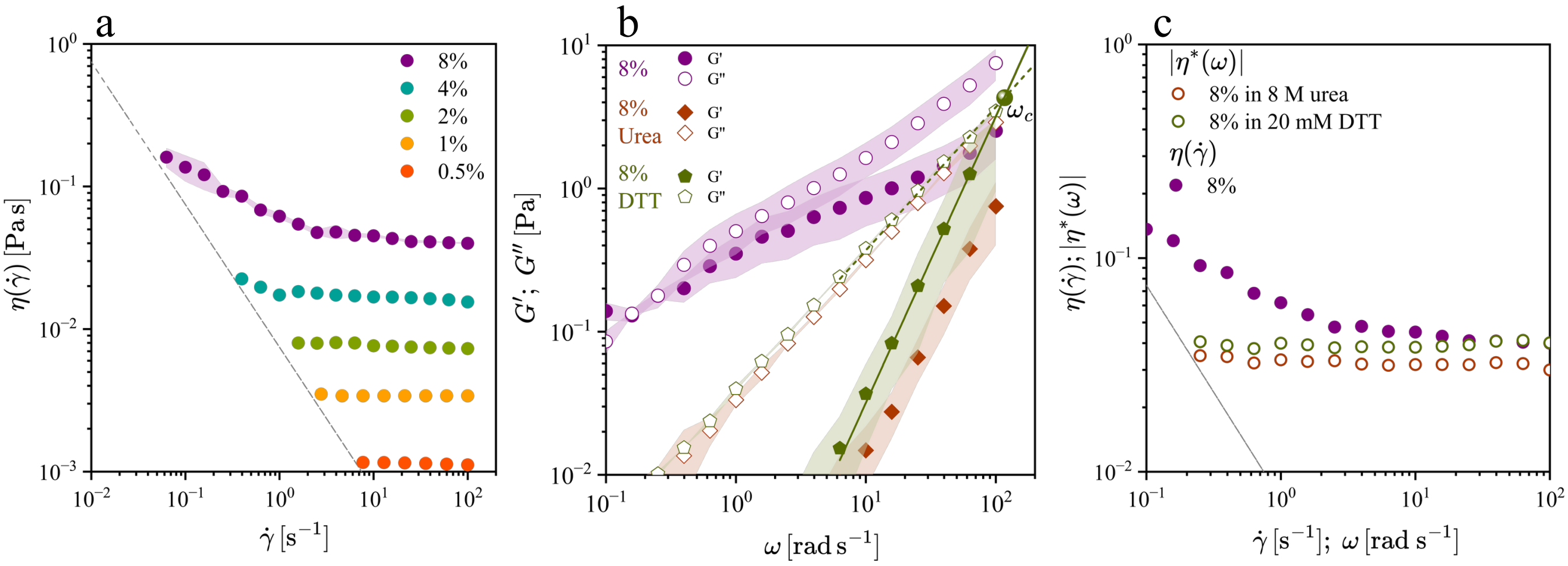

Figure S2 – (a) Steady-shear viscosity as a function of shear rate, measured using a 40 mm cone-and-plate geometry (2° CP-40 mm). The minimum rheometer sensitivity is shown as a grey dashed line. BSM samples at 8, 4, 2, 1, and 0.5 wt% were prepared in 10 mM HEPES buffer at pH 7.0. Consistent with the emergence of a strong concentration-dependent linear viscoelastic response, these steady shear viscosity measurements reveal pronounced shear-thinning behavior for 8 wt% BSM. (b) Viscoelastic behavior of 8 wt% BSM in the absence or in the presence of 8 M urea, or 20 mM dithiothreitol (DTT). Shaded regions represent the standard deviations of three independent measurements. In the presence of DTT, which reduces disulfide bonds between mucin subunits, the covalent network at 8 wt% is disrupted, restoring a terminal liquid-like response with $G' \sim \omega^2$ and $G'' \sim \omega$, consistent with the Maxwell description. The corresponding relaxation time is $\tau = 8.60$ ms. In contrast, 8 wt% BSM in the presence of 8 M urea, which perturbs hydrogen bonding and weak non-covalent associations without reducing disulfide linkages, does not recover terminal behaviour of a viscoelastic liquid: while $G'' \sim \omega$ is preserved, the elastic modulus G' exhibits an apparent $G' \sim \omega^{1.5}$ scaling over the accessible frequency window. (c) Steady-shear viscosity of the untreated 8 wt% BSM sample shown as a function of shear rate, compared with the complex viscosity extracted from G' and G'', shown as a function of angular frequency, for 8 wt% BSM in the presence of either 8 M urea or 20 mM DTT.

For (b) and (c), the context of urea- and DTT-induced denaturation is different: while a small amount of DTT (20 mM) is sufficient to reduce covalent disulfide bonds, 8 M urea alone corresponds to approximately 48% w/v of the final sample. Therefore, urea-induced perturbations represent a major change in the solvent environment. The slightly lower network elasticity in 8 M urea compared to that in 20 mM DTT suggests that the residual viscous response is not controlled simply by the viscosity of the solvent but instead reflects the large perturbation of the solvent environment. This demonstrates how urea can suppress a broader set of non-covalent interactions, leading to a lower residual complex viscosity for the sample in the presence of urea.

For all steady shear or small amplitude oscillatory measurements, interfacial effects and evaporation were avoided by using a 2 vol% Tween 80 layer and a mineral oil overlay, respectively, deposited at the air-liquid interface prior to measurement.

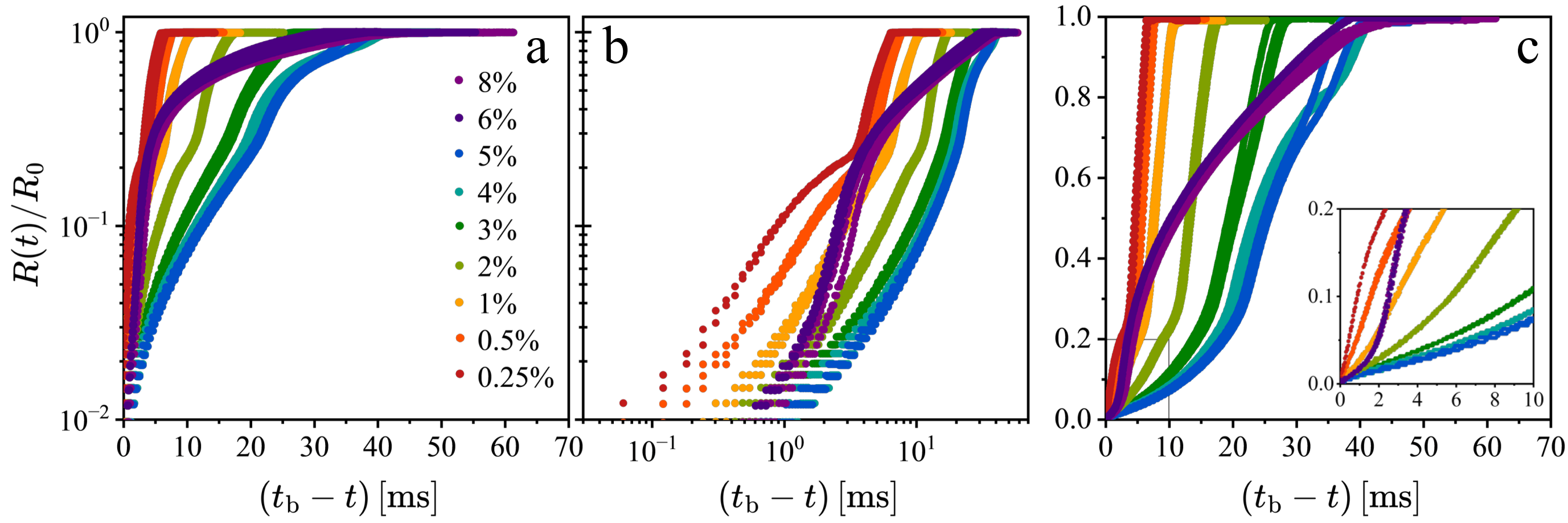

Figure S3 – Plots of the normalized filament radius $R(t)/R_0$ as a function of $(t_b - t)$, where t_b is the time to breakup. For each concentration, at least three independent videos are included. Independent measurements performed using a second high-speed imaging setup (operating at an image acquisition rate of $f_s = 2{,}095$ Hz) and separately prepared BSM solutions reproduced the same concentration-dependent thinning behavior. In the terminal stage of the filament lifetime, replicates at a given concentration superimpose, and the trajectories for all concentrations converge as the filament approaches breakup. For completeness, data are presented using (a) log-linear, (b) log-log, and (c) linear-linear axes. The inset in (c) shows the terminal region on linear-linear axes.

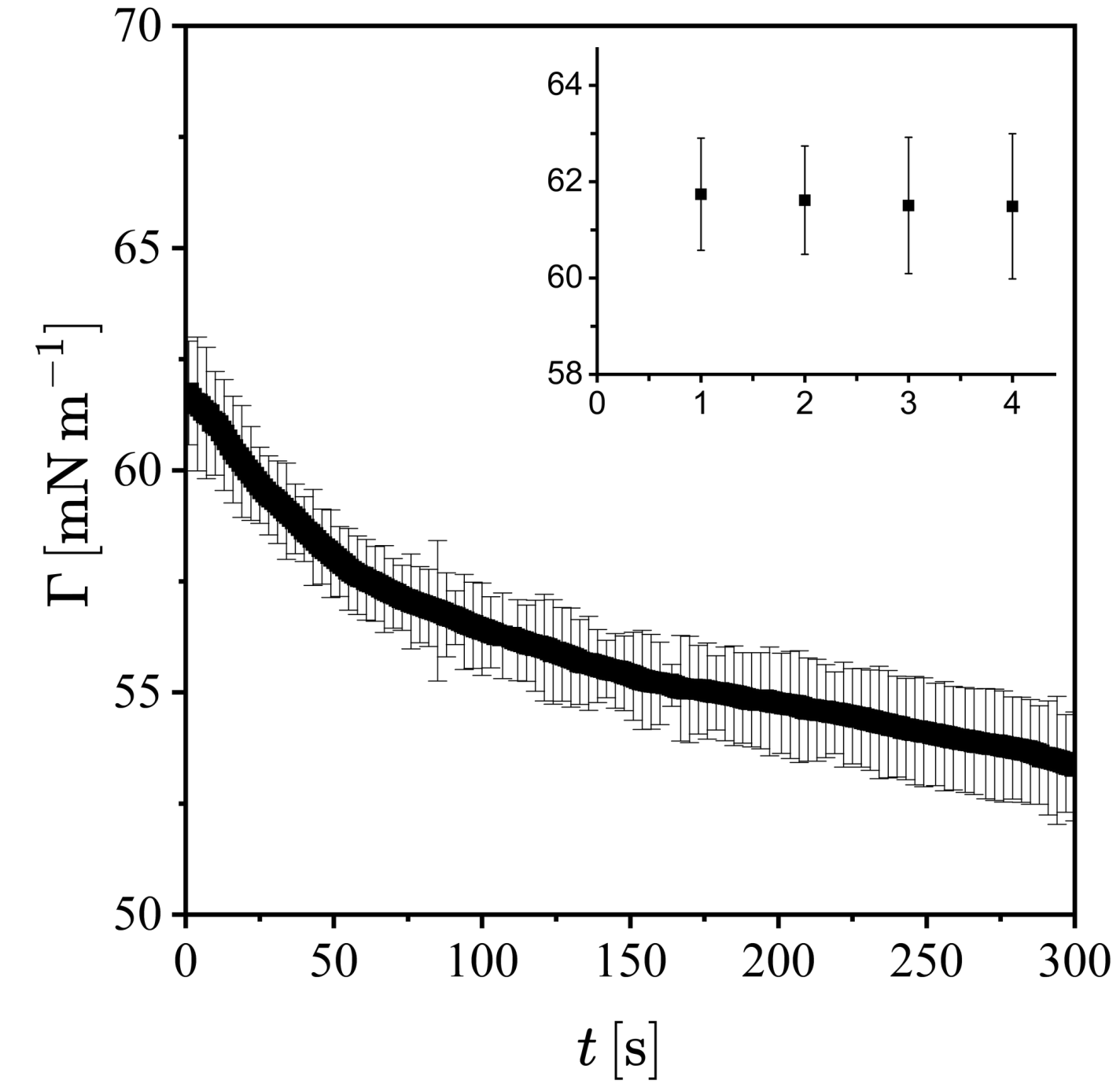

Figure S4 – Surface tension (Γ, mN m^{-1}) of BSM at 2 wt% as a function of time (s). Mean ± standard deviation from triplicate measurements is shown. Across all concentrations (0.25-8 wt%), the initial surface tension is indistinguishable within experimental resolution. Deviations only emerge at longer timescales, beyond the temporal window relevant to DoS measurements (< 1 s). Inset: variation of surface tension over 1-4 s, highlighting negligible early-time variations.

Terminal capillarity-driven thinning behavior of dilute BSM samples

For the dilute BSM samples (Figure S5d), the capillary thinning dynamics at later stages were additionally examined in the $(t_b - t)$ representation, where $t_b - t$ represents the distance from the breakup singularity, to assess whether the filament approaches an asymptotic viscous response close to breakup. In this limit, the minimum filament radius may be written in the general form[2]

$$\frac{R(t)}{R_0} = \frac{2X-1}{2}\left(\frac{t_b-t}{T_\infty}\right),$$

where R_0 is the initial filament radius, t_b is the breakup time, X is a geometric factor associated with the local filament shape, and T_∞ is a characteristic terminal thinning timescale defined as $T_\infty = \eta_{E,\infty} R_0/\Gamma$. Here, $\eta_{E,\infty}$ denotes the asymptotic transient tensile stress growth coefficient and Γ the surface tension. For an approximately cylindrical filament, $X \approx 1$, such that the above expression reduces to

$$\frac{R(t)}{R_0} \approx \frac{1}{2}\left(\frac{t_b-t}{T_\infty}\right).$$

Thus, if the terminal thinning regime is governed by a time-independent asymptotic transient tensile stress growth coefficient, $R(t)/R_0$ should vary linearly with $(t_b - t)$. On a log-log plot, this corresponds to a slope of unity, $R(t)/R_0 \sim (t_b - t)^1$. The late-time data for 0.25 and 0.5 wt% BSM samples are consistent with this scaling, supporting the interpretation of a terminal response in the final stage of thinning (see Figure S5d). Assuming $X \approx 1$, the terminal thinning regime was fitted in the interval $10^{-1} \leq (t_b - t) \leq 10^0$ [ms] using the form $R(t)/R_0 = A(t_b - t)$, where A is the fitted prefactor and the slope was fixed to unity.

Comparison with the asymptotic expression above gives $A = 1/2T_\infty$, so that $T_\infty = 1/2A$ and hence $\eta_{E,\infty} = \Gamma T_\infty/R_0 = \Gamma/2AR_0$. Using $R_0 = 0.635$ mm, and $\Gamma = 61.7$ mN m^{-1}, the fitted prefactor for the 0.5 wt% BSM sample $A = 63.57$ s^{-1} gives $T_\infty = 7.87 \times 10^{-3}$ s and $\eta_{E,\infty} = 0.77$ Pa s. From Figure S2a, the steady shear viscosity was measured to be $\eta_0 = 1.21 \times 10^{-3}$ Pa s, and the Trouton ratio for this semidilute polymer solution is thus $Tr = \eta_{E,\infty}/\eta_0 = 636$. Likewise, for the 0.25% BSM sample, $A = 115.5$ s^{-1}, giving $T_\infty = 4.33 \times 10^{-3}$ s and $\eta_{E,\infty} = 0.42$ Pa s. These asymptotic transient tensile stress growth coefficients obtained close to filament breakup of the dilute BSM samples are consistent with the corresponding apparent transient tensile stress growth coefficient values for the low concentration fluids shown in Figure S6, which may indicate that the dilute samples approach a terminal finite-extensibility dominated thinning regime prior to breakup.

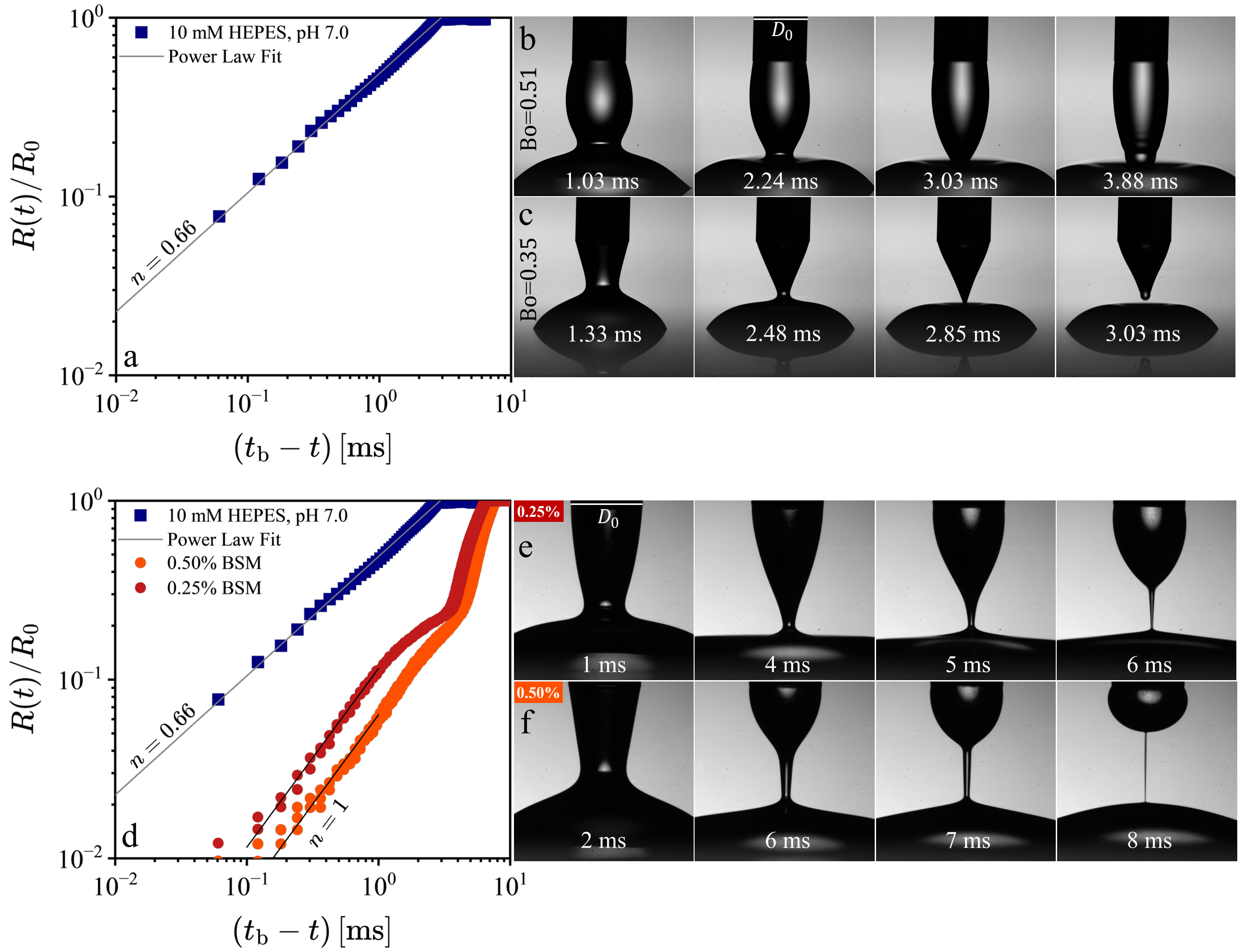

Figure S5 – Dripping-on-substrate measurements with $D_0 = 2R_0 = 1.27$ mm (a) for the solvent used in this work [10 mM HEPES buffer at pH 7.0 (blue)]. The data for 10 mM HEPES buffer at pH 7.0 are shown for the profile extracted from images performed at a Bond number $Bo = 0.35$. Snapshots of 10 mM HEPES buffer at pH 7.0 at a separation distance that yield Bond numbers of (b) $Bo = 0.51$ and (c) $Bo = 0.35$. The conical shape is most clearly observed at smaller needle-substrate separation distances H, corresponding to lower Bond numbers ($Bo = R_0 H \rho g / \Gamma$), as gravitational effects are reduced relative to capillarity at the moment the pendant drop touches the substrate. For the buffer, the Ohnesorge number $Oh = \eta_0 / \sqrt{\rho \Gamma R_0}$ is $Oh \approx 4.4 \times 10^{-3}$, placing the system well within the inertio-capillary regime. The time to breakup (t_b) for HEPES buffer is 2.78 ms. (d) Normalized minimum filament radius $R(t)/R_0$ as a function of $t_b - t$ for 0.25 and 0.5 wt% BSM. In the final stage of thinning, both samples approach $R(t)/R_0 \sim (t_b - t)^1$ as indicated by the slope $n = 1$, consistent with a viscosity-dominated terminal thinning regime. (e-f) Corresponding high-speed image sequences for (e) 0.25 wt% ($t_b = 6.24$ ms) and (f) 0.5% BSM ($t_b = 8.11$ ms).

Filament capture rate (FCR) analysis

The filament capture rate (FCR), defined as $\mathrm{FCR} = f_s \ln(m)$,[3] where m is the spatial dynamic range, is a figure of merit for filament thinning experiments that reflects the resolution of the EC regime and, equivalently, the largest extensional strain rate that can be reliably measured. In this work, FCR varied between 67,700 and 74,400 s^{-1} for the imaging conditions used to visualize this concentration range (2-5 wt%), indicating that effective values of τ_E can be reliably extracted from the observations of EC regime. It is evident from Figure 2b (main text) that for the 6 and 8 wt% BSM concentrations, the thinning of the filament is qualitatively different to that observed at lower concentrations. The extensional strain rate $\dot{\varepsilon}(t)$ increases monotonically with time and the filament radius decreases approximately linearly in time for the first 30 ms, consistent with VC thinning. The subsequent elastocapillary thinning regime is markedly short-lived compared to lower concentrations. FCR values of 52,100 and 56,800 s^{-1} are obtained for 6 and 8 wt% BSM, respectively. Despite the smaller transition radius at the onset of the EC regime and the short period of time for which an elastocapillary balance is established, the filament capture rate for our DoS system is large enough to accurately resolve the thinning dynamics (Figure 2c, h, main text) and determine the corresponding extensional relaxation time. The high filament capture rates (FCR > 52,100 s^{-1}) of our DoS instrument provide very high spatial and temporal resolution, which enables the direct measurement of the extensional relaxation time τ_E for all semi-dilute and concentrated mucin samples studied here.

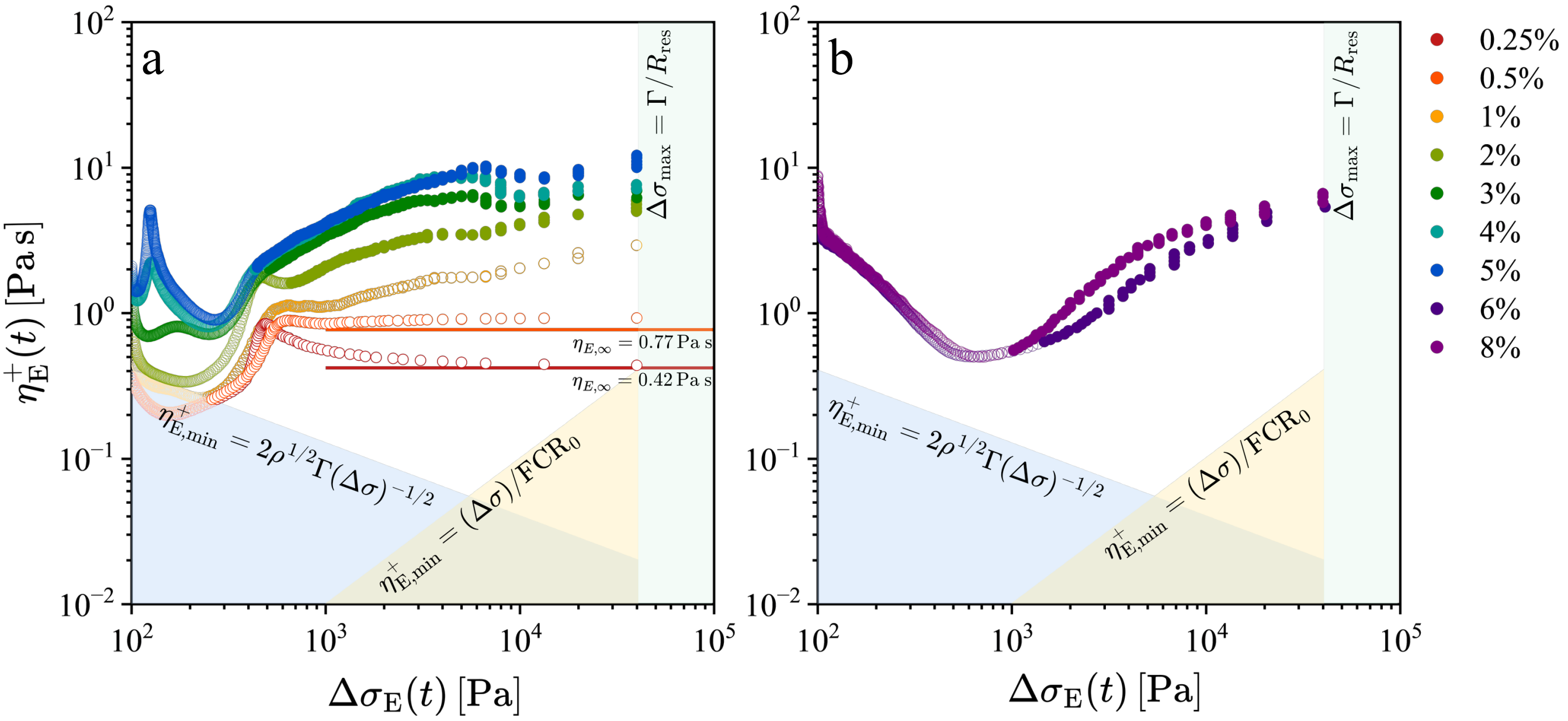

Figure S6 – Apparent transient tensile stress growth coefficient plotted as a function of the time-evolving extensional stress difference for BSM dispersions spanning 1 to 8 wt%. Limit lines, as discussed by Warwaruk *et al.*[3], are drawn using $\rho = 1080$ kg m^{-3}, $\Gamma = 61.7$ mN m^{-1}, $R_0 = 0.635$ mm, $R_{res} = 1$ pixel $= 1.51 \times 10^{-6}$ m, $FCR_0 = f_s \ln m_0 \approx 1.0 \times 10^5$ s^{-1}, with $f_s = 16500$ s^{-1} and $m_0 = R_0/R_{res} = 430.46$. The horizontal lines shown in (a) indicate the asymptotic values of the apparent transient tensile stress growth coefficient asymptotic transient $\eta_{E,\infty}$, as previously discussed (see the discussion on page S-12).

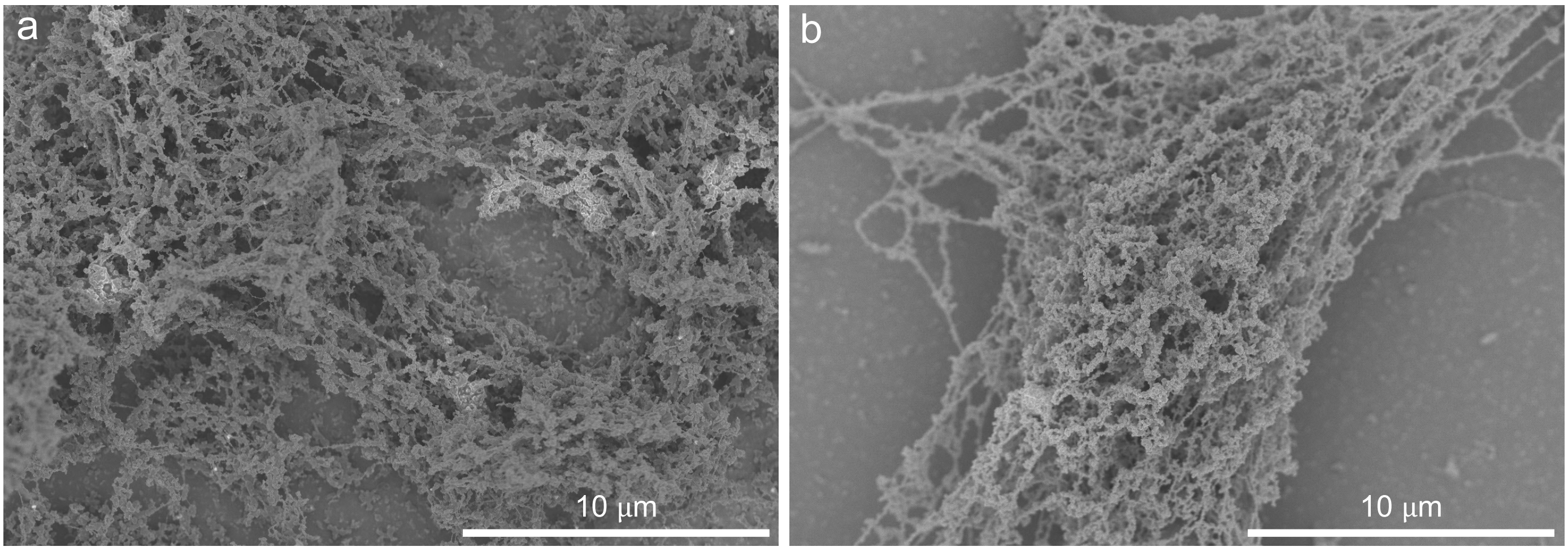

Figure S7 – Representative Scanning Electron Microscopy images of BSM samples prepared at (a) 6 wt% and (b) 5 wt%. Scale bars represent 10 μm.

Table S4 – Measured surface tension using a pendant drop tensiometer (OCA 25, DataPhysics Instruments, Germany). Drops (22 μL) were formed at the tip of a 1.65 mm DS500 GT syringe, and the liquid-air surface tension was obtained by fitting the drop profile to the Young-Laplace equation using dpiMAX software. A density of 1080 kg m^{-3} was assumed in the fitting procedure. Measurements were performed in triplicate for each concentration; the reported value include the standard deviation. The initial surface tension was used in all capillarity-driven thinning analyses, as filament thinning occurs on timescales much shorter than those required for interfacial equilibration.

BSM concentration (wt%)	Surface tension (mN m^{-1})
0.25-8 wt%	61.7 ± 2.3

Table S5 – Reference Ohnesorge number ($Oh = \eta_0/\sqrt{\rho \Gamma R_0}$) for the BSM concentrations investigated. Here, $\rho = 1080$ kg m^{-3}, $\Gamma = 61.7$ mN m^{-1}, $R_0 = 0.635$ mm. The reported value of η_0 is an estimate from the low-shear viscosity as the zero-shear plateau was not directly resolved in steady shear.

BSM concentration (wt%)	η_0 / Pa s	Oh
0.25	0.0010	0.005
0.5	0.0012	0.006
1	0.0035	0.017
2	0.0079	0.038
4	0.0225	0.109
8	0.1604	0.780

Table S6 – Comparison between relaxation times (τ) obtained from SAOS estimated from the extrapolated crossover frequency for 2-5 wt% (and from $\tau = (\mathbb{V}/\mathbb{G})^{1/(1-\beta)}$ at 8 wt%), and the effective relaxation times extracted from elastocapillary (EC) thinning regimes in DoS extensional experiments (τ_E) for BSM across concentrations. The time to breakup (t_b) for each fluid is also shown.

BSM concentration (wt%)	τ (SAOS) / ms	τ_E (DoS) / ms	t_b / ms
0.25	-	-	6.24 ± 0.3
0.5	-	-	8.11 ± 0.2
1	-	0.53 ± 0.08	12.2 ± 0.6
2	1.6	1.34 ± 0.05	18.2 ± 0.5
3	-	2.4 ± 0.08	28.4 ± 1.4
4	2.7	3.0 ± 0.26	39.7 ± 1.9
5	4.3	2.8 ± 0.15	41.5 ± 0.3
6	critical gel	0.34 ± 0.02	42.8 ± 2.5
8	36.4	0.33 ± 0.05	47.1 ± 3.1

References

(1) Song, J.; Holten-Andersen, N.; McKinley, G. H. Non-Maxwellian viscoelastic stress relaxations in soft matter. *Soft Matter* **2023**, *19* (41), 7885–7906. DOI: 10.1039/d3sm00736g.
(2) Dinic, J.; Sharma, V. Macromolecular Relaxation, Strain, and Extensibility Determine Elastocapillary Thinning and Extensional Viscosity of Polymer Solutions. *PNAS* **2019**, *116* (8), 8766–8774. DOI: 10.1073/pnas.1820277116.
(3) Warwaruk, L.; Zinelis, K.; Ewoldt, R. H.; Macosko, C. W.; McKinley, G. H. Dripping-Onto-Substrate (DoS): Dos and Don'ts. *Journal of Rheology* **2026**, *70* (5), 1045–1071. DOI: 10.1122/8.0001163.
